# 

**Published:** 1882-06

**Authors:** 


					﻿THE INTERNATIONAL HAHNEMANNIAN ASSOCIA-
TION.
The second annual meeting of the Association will be held at
the office of J. R. Haynes, M. D., 120 N. Meridian Street, Indian-
apolis, on Tuesday, June 13th, at 3 o’clock p. m.
C. Pearson, Committee.
NOTICE. .	■	/
All articles for publication, books for review, business communications, etc.,
must be sent to Editor, 2109 Chestnut Street, Philadelphia.
CONTRIBUTORS:
Drs. H. 0. Allen, T. F. Allen, Edward Bayard, J. B. Bell, E. W. Berridge,
Geo. Id. Clark, A. C.Cowperthwaite, Wm. J. Guernsey, W. A. Hawley,
John Hall, W. M. James, W. H. Jenny, Ad. Lippe, 0. Lippe,
Malcolm MacFarlan, C. F. Nichols, C. Pearson, T. F. Poin-
- eroy, Leila A. KenDell, G.Carleton Smith, P. P. Wells,
Wm. P. Wesselhoeft, David Wilson (London),
T. P. Wilson, and many others.
Authors of accepted papers are furnished with copies of the
number in which their articles appearapplication for such copies
to be made when sending papers. Any irregularity in the receipt
of this Journal should be promptly reported to Editor.
				

